# Hematobium schistosomiasis control for health management of labor force generation at Nkhotakota and Lilongwe in the Republic of Malawi—assumed to be related to occupational risk

**DOI:** 10.1186/s41182-019-0155-8

**Published:** 2019-05-02

**Authors:** Nobuyuki Mishima, Samuel K. Jemu, Tomoaki Kuroda, Koichiro Tabuchi, Andrew W. Darcy, Takaki Shimono, Pheophet Lamaningao, Mari Miyake, Seiji Kanda, Susan Ng’ambi, Yoshihiro Komai, Hirofumi Maeba, Hiroyuki Amano, Toshimasa Nishiyama

**Affiliations:** 1 Department of Hygiene and Public Health, Kansai Medical University, 2-5-1 Shinmachi, Hirakata, Osaka, 573-1010 Japan; 2 Center for Travel Medicine, Kansai Medical University Medical Center, Osaka, Japan; 3 Department of Urology, Kansai Medical University, Osaka, Japan; 4 Maeba Clinic, Osaka, Japan; 5 Community Health Science Unit, Ministry of Health, Lilongwe, Malawi; 6 Kamuzu Central Hospital, Lilongwe, Malawi

**Keywords:** Hematobium schistosomiasis, Occupational risk, Labor health management, Economic growth, Urine reagent strips, Malawi

## Abstract

**Background:**

In Malawi, hematobium schistosomiasis is highly endemic. According to previous studies, countermeasures have been conducted mainly in school-aged children. In this study, we focused on the age groups, which are assumed to be major labor force generation. Hematobium schistosomiasis is supposed to be related to occupational activities in schistosome-endemic countries because of its infectious route. We chronologically followed the transition of schistosome egg-positive prevalence before and after mass drug administration of praziquantel (MDA) by using a urine filtering examination. We also analyzed the effectiveness of urine reagent strips from the cost perspective.

**Results:**

The egg-positive prevalence was 34.3% (95% CI 28.5–40.5) just before MDA in June 2010 and the highest prevalence was in the age of twenties. The egg-positive prevalence reduced to 12.7% (95% CI 9.2–17.3, *p* < 0.01) 8 weeks after the first MDA and the prevalence reduced to 6.9% (95% CI 4.6–10.0, *p* < 0.01) after the second MDA in August 2011. The egg-positive prevalence after MDA in 2013 was reduced from 3.8% (95% CI 2.1–6.9) to 0.9% (95% CI 0.3–3.4) and *p* value was 0.050. Using urine reagent strips after MDA, the positive predictive value decreased, but the negative predictive value remained high. The cost of one urine reagent strip and one tablet of praziquantel were US$0.06 and US$0.125 in 2013 in Malawi. If the egg-positive prevalence is 40%, screening subjects for MDA using urine reagent strips, the cost reduction can be estimated to be about 24%, showing an overall cost reduction.

**Conclusions:**

MDA of praziquantel can assuredly reduce schistosome egg-positive prevalence. The combination of MDA and urine reagent strips could be both a practical and cost-effective countermeasure for hematobium schistosomiasis. It is key to recognize that hematobium schistosomiasis could be considered a disease that is assumed to have some concern with occupational risk at Nkhotakota and Lilongwe in Malawi. From this point of view, it is very important to manage workers’ health; the sound labor force generation is vital for economic growth and development in these areas and countries.

## Introduction

Schistosomiasis, a trematode infectious disease, is widely distributed around the tropics and subtropics. This infectious disease is one of the world’s three major parasitic infections. It is endemic in 74 developing countries; and approximately 800 million people are at risk of schistosome infection. More than 300 million people suffer from associated severe morbidity [1]. Chronic and repeat infection of schistosomiasis could result in irreversible damage to body organs and other diseases; for example, *Schistosoma haematobium* infection may lead to bladder cancer and cervical cancer [2, 3]. In the schistosome endemic regions, the most prevalent form of the disease is chronic schistosomiasis, resulting from repeated exposure to infectious cercariae [4]. Schistosomiasis mortality rates rises substantially as age increases [5]. Therefore, it is important for healthcare systems to consider not only children but also young adults—assumed to be a major component of labor generation—as the subjects of schistosomiasis control as related to occupational risk.

Schistosomiasis is recognized as one of the neglected tropical diseases (NTDs) at present. Global coverage rate of preventive chemotherapy against schistosomiasis is still low at 8.3%, while the rate against onchocerciasis is 59.8% [1]. In sub-Saharan Africa, approximately 280,000 annual deaths have been attributed to schistosomiasis [6]. Countermeasures have been globally to fight malaria, tuberculosis, and HIV infection; however, we consider provisions against schistosomiasis are an important next step for the sustainable growth and development in countries affected by schistosomiasis because of the burden the disease places on those living in the affected region. This disease does not only cause immediate morbidity in children, but it also has long-term health effects on the children’s development into adulthood. Although the earlier studies targeted school-aged children, but the disease may become a matters of concern for the general public if the overall labor force suffers from schistosomiasis.

Malawi is one of the poorest countries in the world and was ranked as the ninth poorest country in terms of GDP per capita in 2017 that stood at US$323.82 on the list of World Bank. Malawi is an endemic country of schistosomiasis and schistosomiasis is associated with populations living in poverty in sub-Saharan Africa, including Malawi. In order to ameliorate poverty, it is important to improve the working environment; and managing the health of the working population is very important to establish a stable and sustainable economic environment. From the viewpoint of labor healthcare management, controlling schistosomiasis could be one of the most effective countermeasures for those countries affected by schistosomiasis. There are few researches that have taken measures against schistosomiasis from the viewpoint of occupational risk.

In Malawi, the main pathogens are *Schistosoma haematobium* and *S. mansoni*. Schistosomiasis is transmitted through contact with infected freshwater in which these intermediate hosts live. The two intermediate hosts distribute simultaneously. *Bulinus globosus*, the main intermediate host, is distributed all over the country—especially where there are sources of freshwater, such as sugarcane plantations, rice growing schemes, man-made dams, rivers, and ponds. While the genus *Biomphalaria* occurs in Lilongwe and the Linthipe Plain, Chapanaga area in Chinkhwawa District; some parts of Ntchisi, Salima, Karonga, Namwera in Mangochi, and Blantyre. It is reported that schistosomiasis is more rampant in poor rural communities especially places where fishing and agricultural activities are dominant [7]. The pathway of schistosome transmission notably affects farmers, fishermen, irrigation workers, and those whose daily activities involve contact with infested freshwater. Contact with freshwater is the inextricable part of the daily activities of many inhabitants in the area. Malawi is predominantly an agricultural country, and agriculture accounts for about 35% of GDP. Moreover agricultural activities provide more than 80% of the employment in this country [8]; therefore, the vast majority of the population is routinely exposed to the possibility of schistosome infection while working. As a result of this situation, we need to recognize that there could be the occupational risk in suffering from schistosomiasis. The prevalence of the disease in the country is estimated between 40 and 50%; school-aged children are a highly infected group and are intensely affected [9]. It was previously reported that although all sections of the population in the endemic areas can be infected with schistosomiasis, the most vulnerable groups are pre-school (under 5 years old) and school-aged children, adolescent girls, and women of childbearing age [10, 11]. Hematobium schistosomiasis is likely to impact child growth and possibly can cause anemia in all age groups; this would call for the inclusion of the entire populations into future control programs [12].

In this study, which looks at schistosomiasis that could have relation to occupational risk, we targeted residents of all generations, including major segments of the labor force to check the current status of residents in our surveyed areas using mass drug administration of praziquantel (MDA) and urinalysis. Managing the health of the labor force can be expected to contribute to the economic growth and development of Nkhotakota and Lilongwe in Malawi. Findings from our study should also help other tropical and subtropical schistosome-endemic countries.

## Methods

### Study area and population

A survey was conducted in 12 contiguous villages with similar socio-economic and cultural characteristics in Nkhota-District located on the shores of Lake Malawi from June 2010 to August 2011. There were 2043 residents who lived in Nkhotakota in June 2010. Among the residents, there were 1810 people who were more than 4 years old as the subjects for praziquantel administration. The inhabitants of these villages were predominantly subsistence farmers of maize and rice and river/swamp fishermen. The communities had one primary school and functional boreholes. The people can access a healthcare center by walking a few hours. Schools provided health education for the prevention of schistosomiasis.

In 2012–2013, a second survey was conducted. Four target areas in the Lilongwe District were selected where previously we had conducted mass drug administration of praziquantel for all the residents more than 4 years old in 2012 with the cooperation of the Malawi Ministry of Health (Community Health Science Unit). These four target areas were Chisindo, Mtika, Mapiri, and Chisaka in Lilongwe. There were 1393 residents who lived at these four areas in Lilongwe District in June 2013. The target areas were located near the capital Lilongwe, the basic infrastructure was being developed to some extent. Health education for schistosome infection had been provided at the schools. Information about schistosomiasis was also provided via broadcast media.

In analyzing the results, we have grouped the participants in the following age groups: < 4, 5–9, 10–14, 15–19 then 20–29, 30–39 and > 40 years. The dosage of praziquantel was determined by body weight, and then we needed to assume a change in body weight under the age of 20, so we divided the age every 5 years.

### Urine examination survey

A list of registered inhabitants (1810 people) was prepared by a door-to-door survey in April 2010. The chief staff of Community Health Science Unit belonging to Ministry of Health conducted the recruitment of participants with the cooperation of the Kamuzu Hospital and all family heads in every district, using printed literature written in Chichewa (Malawian domestic language) that included information on the aims of the study. In reference to the previous study of the Malawi national survey [8], the sample sizes necessary for assessment of urine examination were set from 250 to 350. The urine examination was conducted for 242 subjects who were more than 4 years old in June 2010, 260 in August 2010, 315 in June 2011, and 350 in August 2011. Total 1167 examinations were conducted through four surveys and urine examinations in each survey were analyzed individually. We distributed instruction on urine examination written in Chichewa to all participants and then they received an explanation on urine examination in Chichewa from the Malawi Ministry of Health staff. After finishing the questions and answers about the explanation more than 1 h, only those who agreed to the examination were selected as the participants. We got the informed consents in writing by them. The participants provided their urine samples and with a urine test administered among the subjects.

In 2013, the recruitment of participants was conducted as same as done in 2010. The sample sizes necessary for assessment of urine examination were set from 200 to 300 as in 2010–2011. Urine examination was conducted for 264 subjects in June 2013 and 211 in August 2013. We examined 475 urine samples provided from all participants, regardless of age.

The urinalysis was performed twice in a year; the first was done immediately before mass drug administration of praziquantel, and the second analysis was performed 8 weeks after the administration [13]. The freshly passed mid-day urine samples were collected from 10 am to 2 pm and were screened for microhematuria and proteinuria using urine reagent strips (SD UroColor 11, Standard Diagnostics Inc., Korea). The urine reagent strips were used according to the manufacturer’s instructions, and all strips were checked in about 30 s. The specimens were then processed for microscopic examination of schistosome eggs at the site of the collection within 3 h.

Processing the specimen and egg detecting followed the syringe filtration technique. A urine subsample of 10 mL was drawn into a plastic syringe from each well-mixed sample and strained through a nylon filter (12 μm pore size: Disease Control Textiles, Vestergaard Frandsen Group, Denmark). The filter was then examined under the microscope with a magnification of × 100, and *S. haematobium* eggs were detected.

### Mass drug administration

After the urine examinations were completed, we provided the information of praziquantel (E. Merck KG) written in Chichewa to all villagers. They received an explanation on the drug in Chichewa from the Malawi Ministry of Health staff. Then we got the informed consent for administering praziquantel in writing from the participants. In the case of those participants who were under 20 years of age, we also obtained confirmation from their guardians. Concurrently, we checked, in advance, the drug allergy history of all the participants and no one had a praziquantel allergy. Regarding the safety of praziquantel administration, we consider those under the age of 4 years as an ineligible population. Women within their first trimester of pregnancy and those who had a history of epilepsy or other signs of potential neurological disease were also excluded from mass drug administration. Praziquantel 40 mg/kg was administered to eligible participants (838 people in 2010 and 1027 in 2011, 728 in 2013), who agreed by Directory Observed Treatment, Short Course (DOTS) protocol by using weight scales. After praziquantel administration by DOTS, we confirmed whether there were no adverse reactions to each participant for at least 30 min under the supervision of physicians. In addition, the Malawi Ministry of Health staff lectured on the prevention of schistosomiasis to all participants using printed materials written in Chichewa.

## Results

### Data analysis

The quantitative data were analyzed using SPSS (version 20.0.0). First, SPSS was used for cross tabulation and running of frequencies. Secondly, the chi-square test was used to establish the relationship between the categorical variables. A *p* value less than 0.05 was considered statistically significant. Graphs were drawn using Apple Inc. Numbers (Ver.4.3.1).

### Egg-positive prevalence in 2010–2011

**Fig. 1:**
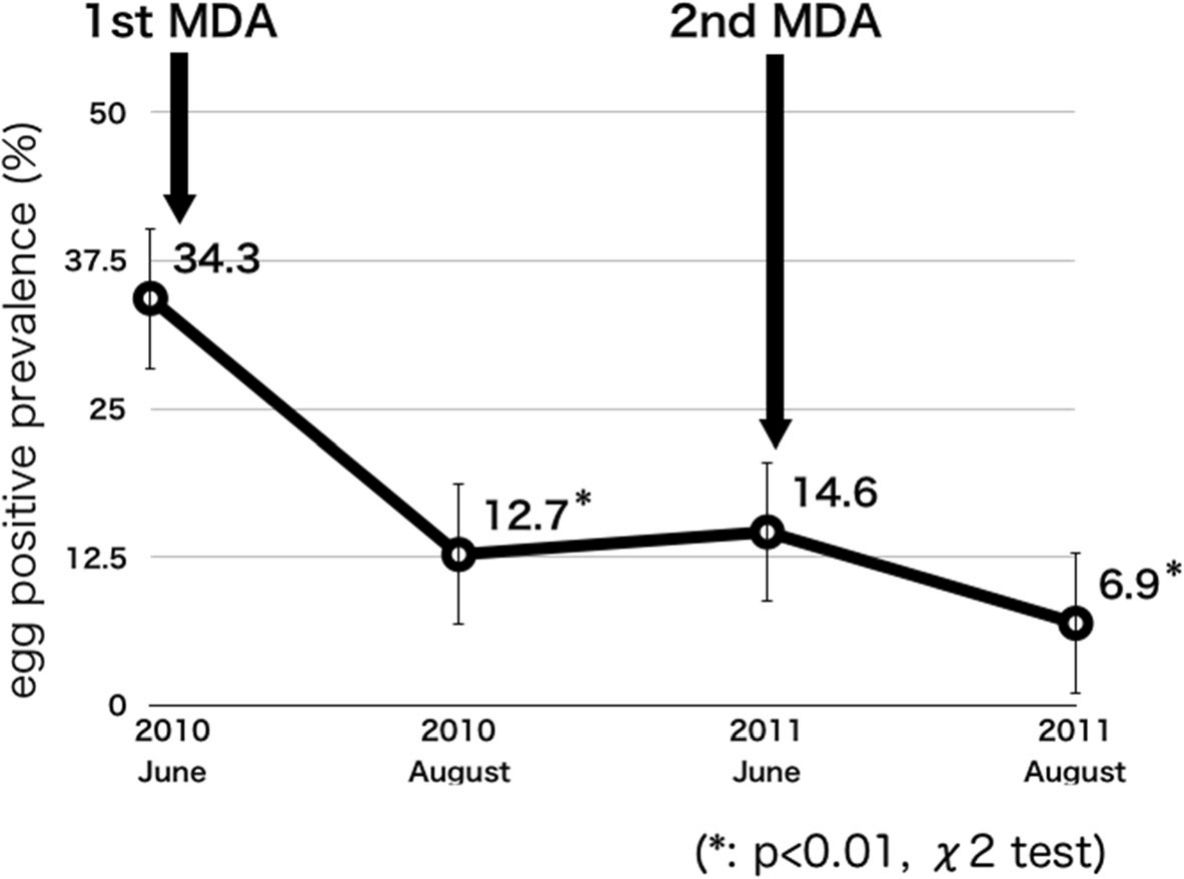
Process of egg-positive rate among all participants 2010–2011

**Fig. 2:**
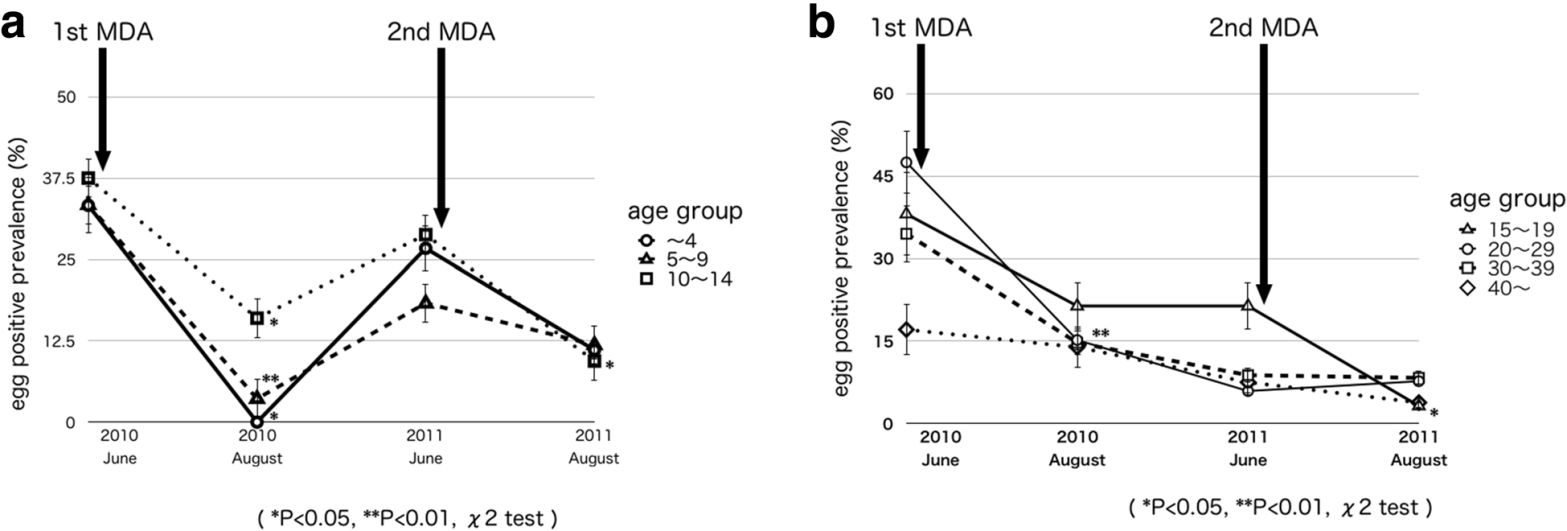
**a** Process of egg-positive rate by age group 2010–2011. **b** Process of egg-positive rate by age group 2010–2011

Table 1 shows the summary of urine filtering examination. The study focused on a random sample of people who participated in our survey: 242 people (age 22.69 ± 35.56 years) in June 2010; 260 people (age 22.81 ± 35.47 years) in August 2010; 315 people (age 23.55 ± 38.00 years) in June 2011; and 350 people (age 23.56 ± 37.57 years) in August 2011. Research showed the egg-positive prevalence for males was 42.6% (95% CI 33.6–52.1) and for female participants was 27.6% (95% CI 20.7–35.8). Figure 1 shows the egg-positive prevalence among all participants in 2010–2011. The schistosome egg-positive prevalence among the participants was 34.3% (95% CI 28.5–40.5) just before mass drug administration (MDA); and this result was similar to the previous report [9]. By age group, the highest egg-positive prevalence was detected in participants in their 20s and it was 47.5% (95% CI 32.8–62.6) before the first MDA, among this group the egg-positive prevalence of males in their 20s was 53.3% (95% CI 29.9–75.4). The first MDA was conducted for 838 participants (46.3%) just after the urine examinations. No one who took praziquantel showed any obvious side effects. The schistosome egg-positive prevalence 8 weeks after MDA was 12.7% (95% CI 9.2–17.3), and it was significantly lower than that before the mass treatment in June (*p* < 0.01). Egg-positive prevalence decreased in all age groups. One year after the first MDA, egg-positive prevalence was 14.6% (95% CI 11.1–19.0) in June 2011 and MDA supposedly kept the prevalence low. However, the prevalence increased in the under-15 age group shown in Fig. 2a, b, those are the process of egg-positive prevalence by age groups in 2010–2011. Among those in the under-15 age group, the gradient of the increasing line of the under-5 group was the steepest. The egg-positive prevalence of those in the under-5 age group increased from 0 to 26.7% 10 months after checking urinalysis in August 2010. On the other hand, the egg-positive prevalence for participants between 15 and 19 years remained the same and the prevalence of those in the older than 20 age group decreased. The second MDA for 1027 participants (56.7%) was conducted just after the urine examination in June 2011, and 8 weeks later the egg-positive prevalence was 6.9% (95%CI 4.6–10.0). The egg-positive prevalence significantly decreased after the second MDA (*p* < 0.01) in August 2011 and the total reduction rate completed sequential MDA was 79.9%. The egg-positive prevalence was significantly reduced among those in the age groups of 10–14-year-olds and 15–19-year-olds (*p* < 0.05). After the second MDA, none of the people who took praziquantel had obvious side effects.

**Table 1:** Results of urine filtering examination 2010-2011

			2010							2011						
			June			August			*P* value	June			August			*P* value
			Number of Subjects	Before 1st MDA		Number of Subjects	8 weeks after 1st MDA			Number of Subjects	Before 2nd MDA		Number of Subjects	8 weeks after 2nd MDA		
				Egg positive number	Egg positive prevalence (95% CI)		Egg positive number	Egg positive prevalence (95% CI)			Egg positive number	Egg positive prevelence (95% CI)		Egg positive number	Egg positive prevalence (95% CI)	
Total			242	83	34.3 (28.5-40.5)	260	33	12.7** (9.2-17.3)	*P*<0.01	315	46	14.6 (11.1-19.0)	350	24	6.9** (4.6-10.0)	*P*<0.01
Male			108	46	42.6 (33.6-52.1)	123	16	13.0** (8.1-20.2)	*P*<0.01	152	25	16.4 (11.4-23.2)	172	9	5.2** (2.8-9.7)	*P*<0.01
Female			134	37	27.6 (20.7-35.8)	137	17	12.4 (8.3-20.0)	*P*<0.01	163	21	12.9 (8.5-19.0)	178	15	8.4 (5.1-13.5)	*P*=0.181
Age group	〜4	Male	5	3	60.0 (22.8-88.4)	6	0	0.0* (0.0-39.5)	*P*<0.05	11	3	27.3 (9.6-56.8)	12	1	8.3 (1.5-35.7)	*P*=0.231
		Female	4	0	0.0 (0.0-49.5)	4	0	0.0 (0.0-49.5)	-	4	1	25.0 (4.5-70.3)	6	1	16.7 (3.0-56.7)	*P*=0.322
		Total	9	3	33.3 (11.9-64.8)	10	0	0.0* (0.0-28.2)	*P*<0.05	15	4	26.7 (10.8-52.2)	18	2	11.1 (3.1-33.1)	*P*=0.249
	5〜9	Male	25	8	32.1 (17.1-51.8)	26	0	0.0 (0.0-13.1)	*P*<0.01	31	5	16.1 (7.0-32.8)	32	2	6.3 (1.7-20.3)	*P*=0.212
		Female	29	10	34.5 (19.8-52.8)	30	2	6.7** (1.7-20.3)	*P*<0.01	29	6	20.7 (9.8-38.6)	27	5	18.5 (8.1-36.9)	*P*=0.838
		Total	54	18	33.4 (22.1-46.8)	56	2	3.6** (1.0-12.2)	*P*<0.h01	60	11	18.3 (10.5-30.1)	59	7	11.9 (5.8-22.7)	*P*=0.325
	10〜14	Male	22	11	50 (30.6-69.4)	24	3	12.5** (4.3-31.2)	*P*<0.01	24	8	33.3 (17.9-53.5)	21	3	14.3 (4.9-34.9)	*P*=0.138
		Female	26	7	26.9 (13.6-46.3)	26	5	19.2 (8.4-38.1)	*P*=0.510	28	7	25.0 (12.6-43.6)	32	2	6.3* (1.7-20.3)	*P*<0.05
		Total	48	18	37.5 (25.1-51.8)	50	8	16.0* (8.3-28.7)	*P*<0.05	52	15	28.8 (18.2-42.4)	53	5	9.4* (4.1-20.4)	*P*<0.05
	15〜19	Male	11	5	45.5 (21.1-72.2)	14	3	21.4 (7.5-47.9)	*P*=0.201	14	2	14.3 (4.0-40.2)	17	0	0.0 (0.0-18.7)	*P*=0.107
		Female	10	3	30.0 (10.7-60.6)	14	3	21.4 (7.5-47.9)	*P*=0.632	14	4	28.6 (11.6-54.9)	14	1	7.1 (1.3-31.8)	*P*=0.139
		Total	21	8	38.1 (20.6-59.3)	28	6	21.4 (10.1-39.7)	*P*=0.201	28	6	21.4 (10.1-39.7)	31	1	3.2* (0.6-16.4)	*P*<0.05
	20〜29	Male	15	8	53.3 (29.9-75.4)	19	4	21.1 (8.4-43.6)	*P*=0.051	21	2	9.5 (2.6-29.2)	23	2	8.7 (2.4-27.0)	*P*=0.924
		Female	25	11	44.0 (26.5-63.1)	27	3	11.1** (3.8-28.3)	*P*<0.01	30	1	3.3 (0.6-16.9)	29	2	6.9 (1.9-22.2)	*P*=0.533
		Total	40	19	47.5 (32.8-62.6)	46	7	15.2 (7.5-28.4)	*P*<0.01	51	3	5.9 (2.0-16.1)	52	4	7.7 (3.0-18.3)	*P*=0.715
	30〜39	Male	14	5	35.7 (16.2-61.5)	16	4	25.0 (10.1-49.7)	*P*=0.523	16	2	12.5 (3.5-36.3)	18	1	5.6 (1.0-26.0)	*P*=0.476
		Female	15	5	33.3 (15.1-58.5)	18	1	5.6* (1.0-26.0)	*P*<0.05	18	1	5.6 (1.0-26.0)	18	2	11.1 (3.1-33.1)	*P*=0.546
		Total	29	10	34.5 (19.8-52.8)	34	5	14.7 (6.4-30.3)	*P*=0.066	34	3	8.8 (3.0-23.1)	36	3	8.3 (2.8-22.0)	*P*=0.942
	40〜	Male	16	6	37.5 (18.3-61.6)	18	2	11.1 (3.1-33.1)	*P*=0.070	24	3	12.5 (4.3-31.2)	25	0	0.0 (0.0-13.6)	*P*=0.068
		Female	25	1	4.0 (0.7-19.8)	18	3	16.7 (5.8-39.5)	*P*=0.158	29	1	3.4 (0.6-17.4)	27	2	7.4 (2.0-23.6)	*P*=0.511
		Total	41	7	17.1 (8.5-31.4)	36	5	13.9 (6.0-28.8)	*P*=0.701	53	4	7.5 (2.9-18.0)	52	2	3.8 (1.0-13.1)	*P*=0.414

(*p<0.05, **p<0.01)

### Urine reagent strips and cost-effectiveness

**Fig. 3:**
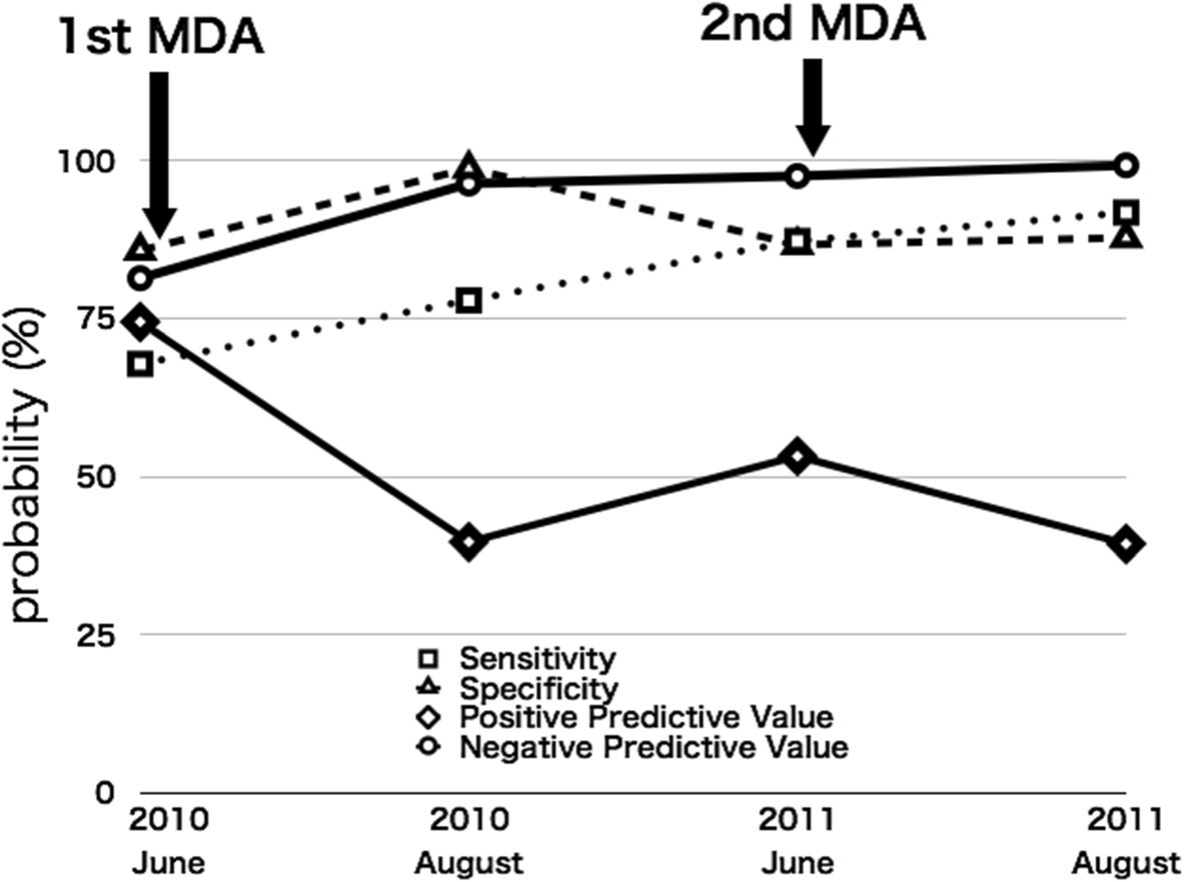
Analysis for the results of occult blood by urine strip tests

Figure 3 shows an analysis for the results of occult blood by the urine reagent strips in 2010–2011. After MDA, the positive predictive value decreased but the negative predictive value remained high, more than 96%. After the second MDA, the negative predictive value was 99.2% (95% CI 98.1–100). The sensitivity was 91.7% (95% CI 80.6–100) and the specificity was 87.7% (95% CI 83.9–91.6).

The cost of one urine reagent strip and one tablet of praziquantel were US$0.06 and US$0.125 in 2013 in Malawi. After MDA in reducing the egg-positive prevalence, the positive predictive value was decreased and the negative predictive value was more than 96%. This suggests that it is practical to exclude urine occult blood-negative subjects from receiving praziquantel.

### Egg-positive prevalence in 2013

**Fig. 4:**
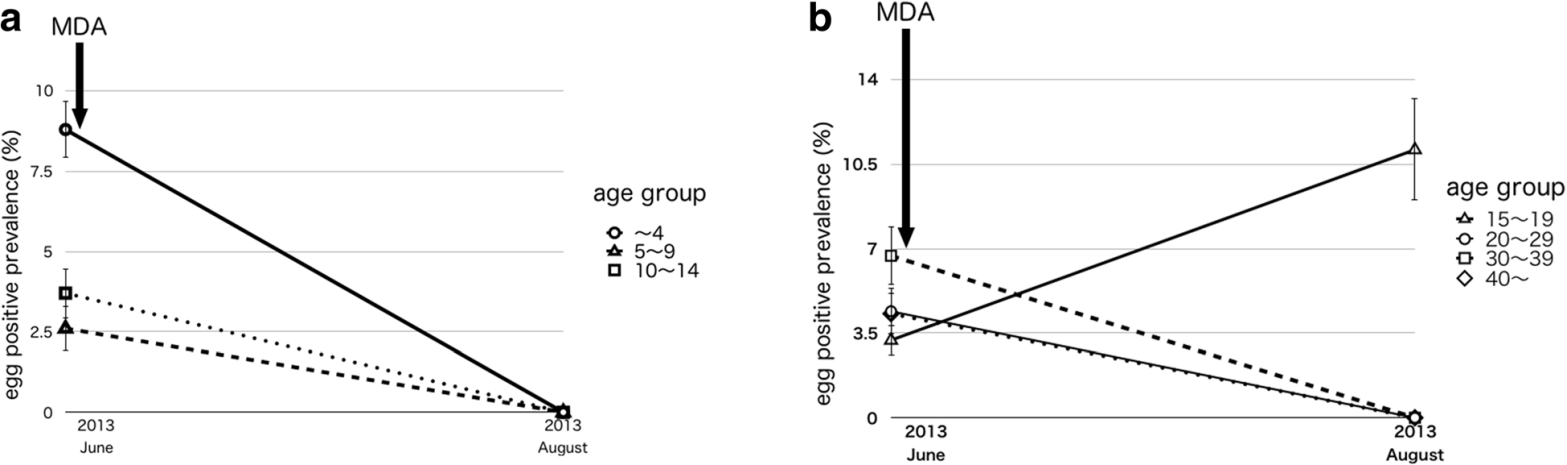
**a** Process of egg-positive rate by age group 2013. **b** Process of egg-positive rate by age group 2013

The surveyed areas in 2013 were the same areas where the MDA of praziquantel was previously conducted in 2012. Table 2 shows that the overall MDA coverage rate in 2012 had ranged from 50.25 to 85.8%. Table 3 shows the result for the urine filtering examination in 2013. Total participants were 264 people (age 21.78 ± 38.42 years) in June and 211 (age 19.99 ± 36.66 years) in August, and they were selected by random sampling among all the residents. The egg-positive prevalence in 264 participants was 3.8% (95% CI 2.1–6.9), it was relatively lower than the average prevalence of the country and the highest was 8.8% (95% CI 3.0–23.1) in those under 5 (Table 3). Eight weeks after MDA, egg-positive prevalence decreased to 0.9% (95% CI 0.3–3.4, *p* = 0.050). The reduction rate was 76.3%. Schistosome eggs were not detected at all in most age groups, excluding the 15–19 age group. Those in the 15–19 age group had an egg-positive prevalence was 11.1% (95% CI 3.3–33.1) 8 weeks after MDA shown in Fig. 4a, b, those are the process of egg-positive prevalence by age groups in 2013. The youngest subject among the egg-positive group was a 2-year-old boy. Praziquantel 40 mg/kg was administered to 728 participants (52.3%) more than 4 years old by DOTS immediately after urine examination. We confirmed that no one experienced any side effects from the praziquantel administration.

**Table 2:** MDA Coverage Rate by Area in 2012

Socio-demographic characteristics area	Proportional MDA attendance by area 2012		
	Registered	Treated	Percentage coverage (%)
Chisindo	390	196	50.3
Mtika	331	284	85.8
Mapiri	296	192	64.9
Chisaka	376	200	53.2
Total	1393	772	55.4

**Table 3:** Results of urine filtering examination 2013

			2013						
			June			August			*P* value
			Number of Subjects	Before MDA*		Number of Subjects	8 weeks after MDA*		
				Egg positive number	Egg positive prevalence (95% CI)		Egg positive number	Egg positive prevalence (95% CI)	
Total			264	10	3.8 (2.1-6.9)	211	2	0.9 (0.3-3.4)	*P*=0.050
Male			99	6	6.1 (2.8-12.7)	86	1	1.2 (0.2-6.4)	*P*=0.082
Female			165	4	2.4 (0.9-6.1)	125	1	0.8 (0.1-4.5)	*P*=0.293
Age group	〜4	Male	14	1	7.1 (1.3-31.8)	8	0	0.0 (0.0-32.9)	*P*=0.439
		Female	20	2	10.0 (2.8-30.4)	15	0	0.0 (0.0-20.7)	*P*=0.207
		Total	34	3	8.8 (3.0-23.1)	23	0	0.0 (0.0-14.6)	*P*=0.143
	5〜9	Male	20	1	5.0 (0.9-23.9)	22	0	0.0 (0.0-15.1)	*P*=0.288
		Female	19	0	0.0 (0.0-17.1)	19	0	0.0 (0.0-17.1)	-
		Total	39	1	2.6 (0.4-13.3)	41	0	0.0 (0.0-8.7)	*P*=0.302
	10〜14	Male	28	1	3.6 (0.6-17.9)	22	0	0.0 (0.0-15.1)	*P*=0.371
		Female	26	1	3.8 (0.7-19.1)	33	0	0.0 (0.0-10.6)	*P*=0.256
		Total	54	2	3.7 (1.0-12.7)	55	0	0.0 (0.0-6.7)	*P*=0.150
	15〜19	Male	9	1	11.1 (2.0-43.9)	8	1	12.5 (2.2-47.5)	*P*=0.929
		Female	22	0	0.0 (0.0-15.1)	10	1	10.0 (1.8-40.8)	*P*=0.132
		Total	31	1	3.2 (0.6-16.4)	18	2	11.1 (3.1-33.1)	*P*=0.267
	20〜29	Male	13	1	7.7 (1.4-33.6)	15	0	0.0 (0.0-20.7)	*P*=0.274
		Female	32	1	3.1 (0.5-15.9)	20	0	0.0 (0.0-16.4)	*P*=0.425
		Total	45	2	4.4 (1.2-15.0)	35	0	0.0 (0.0-10.1)	*P*=0.207
	30〜39	Male	3	1	33.3 (6.1-79.5)	2	0	0.0 (0.0-66.2)	*P*=0.361
		Female	12	0	0.0 (0.0-24.6)	5	0	0.0 (0.0-43.9)	-
		Total	15	1	6.7 (1.2-30.1)	7	0	0.0 (0.0-35.9)	*P*=0.484
	40〜	Male	12	0	0.0 (0.0-24.6)	9	0	0.0 (0.0-30.3)	-
		Female	34	2	5.9 (1.6-19.3)	23	0	0.0 (0.0-14.6)	*P*=0.236
		Total	46	2	4.3 (1.2-14.7)	32	0	0.0 (0.0-10.9)	*P*=0.232

## Discussion

Agriculture is one of the main sources of employment for people in developing countries such as Malawi. In fact, agriculture produces employment for more than 80% of the active labor force in Malawi [7]. The International Labor Organization (ILO) states that agricultural work is one of the most hazardous occupational activities to health worldwide [14]. Many kinds of agricultural activities are related to occupational injuries and those who are engaged in agriculture are being exposed to the risk of schistosome infection because they come into contact with freshwater during farm work in schistosome-endemic countries. Although schistosomiasis is not specifically documented in the list of ILO occupational diseases [15], it should be related to the occupational risks. Schistosomiasis is associated with populations living in poverty in sub-Saharan Africa, including Malawi. In order to ameliorate poverty, it is key to improve the working environment and maintain sustainable economic growth. It is because good health significantly promotes economic growth, both in the short run and long run [16]. The labor force age ranges from 15 to 64 years of age, and 15 to 29-year-olds are presumed to be the main labor force generation, accounting for 51.6% among all labor force generation [17]. If schistosomiasis has occupation-related risks, then adequate healthcare service should be provided for the labor force in order to provide a stable GDP growth rate. Improving health conditions boosts the productivity of workers and that increases economic growth in the longer term [16]. Thus, it is expected that protecting the health of the labor force could lead to a reduction in poverty in tropical and subtropical countries and mobilize national development in the region.

Schistosomiasis is prevalent throughout Malawi. Since the late 1990s, the decline in human capital accelerated the collapse of public health services [18]. The country depends on the income generated from agriculture. More than half of the Malawi population is food insecure [19], and 65.3% of the people are unable to meet their daily dietary needs [20]. According to previous studies [7–10, 21–24], school-aged children are a high-risk group for schistosomiasis infection. Our study showed that before the first MDA in June 2010, those in the twenties showed the highest egg-positive prevalence (Table 1). Those in the 20s are assumed to belong to the labor force. To alleviate poverty, and cases of schistosomiasis related to poverty, it is important for the economic growth and development of this country to protect the health of the labor force. In the older-than-20 age group, the egg-positive prevalence decreased 10 months after the first MDA (Fig. 4b). In general, providing health information about schistosomiasis may bring about behavioral changes in the population that would improve the overall health in the country.

An increase in the egg-positive prevalence was observed in the under-15-age group 1 year after MDA in June 2011. Among the under-15-age group, the gradient of the increasing line of under-5 age-group was steepest (Fig. 2a). Positive egg prevalence of each age group in 2013 was lower than the average in Malawi, and the highest was 8.8% in the under-5-age group (Table 3). There is a high risk of infection for those under the age of 15 years, and it is suggested that this tendency may be greater among those under the age of 5 years. Previous studies reported that pre-school children are also at the risk of schistosome infection [25], and when school-aged children were screened schistosome infection ranged from 5 to 57% [26]. In order to confirm the minimum age of schistosome infection, all subjects, of all ages, in the study underwent a urine examination in 2013. We detected that the age of the youngest infected subject was 2 years old in this study. Pre-school children frequently accompany their guardians into the freshwater areas [21]. Although it is known that pre-school children also face schistosome infection [27], there is still room for further study on the safety of administering praziquantel to children less than 4 years of age. MDA, however, may be a more promising approach to disease control in Malawi [28]. The Malawi National Schistosomiasis Control Program does not have well-documented evidence of universal drug treatment [29]. As the first step in breaking the chain of chronic and repetitive infection, the first year of school enrollment is considered an appropriate time for the first MDA after birth.

In only 15–19 age group, elevation of the egg-positive prevalence was confirmed after MDA in August 2013 (Fig. 4b). Therefore, those who graduated from schools—who are in the 15–19 age group—belong to the high-risk group for repeat schistosome repeated.

Referring to the population pyramid of Malawi in 2010, teens, 20s, and school-aged children accounted for about 60% of the total population [30]. The population of teens and 20s is more than four million. These two generational groups are presumed to be major labor force; so if their health suffers due to schistosome infection, it may have undesirable effects on national development and economic growth. As previously mentioned, agriculture is the primary industry and provides more than 80% of employment in the country [7]. Moreover, community-wide MDA of praziquantel is highly cost-effective when compared with the treatment of school-aged children alone [31]. Therefore, it is important to protect the health of not only school-aged children but also the overall labor force. There seems to be a link between health and income growth in the schistosomiasis endemic areas. The main route of schistosome infection is through contact with infected freshwater. In our target areas, it is considered that local residents are in contact with freshwater through their daily activities such as fishing, farming, washing, bathing, swimming and so on. The occupations which are at risk of infection with schistosomiasis in Malawi are rice farmers, sugarcane growers, irrigators thus those responsible for opening water flow in canals, fishermen, tobacco growers, vegetable farmers, cattle wrestlers, held man, cane cutters, fish pond workers, and wildlife guards [9]. For instance in Japan, a former schistosomiasis endemic country (*Schistosoma japonicum*), schistosomiasis was regarded as an occupational disease for rice farmers [32]. And farmers’ health injuries caused by schistosomiasis were symbolically elaborated in Katayama Memoir (Katayama-ki) written by Dr. Yoshinao Fujii in 1847. Human excrement, often containing schistosome eggs, is spread in fertilizing the fields, and the barelegged farmers in rice-paddies are easy victims for cercariae. Generally, urination and defecation are the main methods for schistosome eggs to get into the environment. The transmission of schistosomiasis in Malawi remains fragmented [33], and setting up proper toilets and designating specific places for latrines/toilets in each district is thought to be an effective method of infection control. From 2010 to 2011 survey, the egg-positive prevalence after MDA decreased, but it was not eradicated. On the other hand, in the survey of 2013, egg-positive prevalence was reduced to 0% in all age groups except in the 15–19 age group (Fig. 4a, b). The 15–19 age group is one of the main labor force groups in this area. It is estimated that this age group also frequently contact freshwater. The surveyed areas in 2013 are located near the Capital City Lilongwe, where health education and promotion about schistosomiasis infection was conducted at schools and there were frequent media broadcasts on the subject. These activities may have led to differences in the survey results. Workers in rural areas are more likely to earn less than their counterparts in urban areas [15]. The difference in income may also have a bearing on schistosome infection. In Nkhotakota District on Lake Malawi, it is assumed that many residents are in contact with fresh water area more frequently because agricultural activities are greater than in the environs of Lilongwe.

Hematobium schistosomiasis can cause and aggravate anemia caused by low dietary iron, hookworm infection, and malaria in Malawi. The health hazards related to hematobium schistosomiasis can include bladder cancer and cervical cancer, but most likely there are repeated infections and chronic infections occurring with these severe diseases. Although bladder cancer occurs principally as urothelial carcinoma, the major histological cell type of bladder cancer associated with hematobium schistosomiasis is squamous cell carcinoma [34–36]. Because the occurrence of squamous cell carcinoma is associated with persistent chronic inflammation, it is essential to prevent chronic infections and repetitive infections to suppress its development.

MDA of praziquantel was conducted in June 2010, and the egg-positive prevalence 1 year later decreased to about two-fifths as shown in Fig. 1 (*p* < 0.01). Egg-positive prevalence in the target areas in 2013 where MDA was conducted in 2012 was 3.8%; and it was supposed to be lower than previously reported egg-positive prevalence in Malawi. These results showed that MDA of praziquantel could certainly reduce the egg-positive prevalence of hematobium schistosomiasis. Although MDA is not an effective replacement for the existing vector control, MDA has the potential to reduce transmission for a limited time and has to be repeated regularly for sustained effect [37]. However, the egg-positive prevalence in August 2013 was reduced from 3.8 to 0.9% 2 months after MDA (*p* = 0.050). This result indicates that the effectiveness of annual MDA might reduce over time. Thus, it may not be necessary to carry out MDA every year. After mass drug administration of praziquantel, no resident showed serious adverse reactions and is also considered safe and appropriate.

Former studies indicated the cost-effectiveness of urine reagent strips [38, 39], but it is still uncertain whether the urine reagent strip is cost-effective [40, 41]. We analyzed the effectiveness of the urine reagent strips for checking hematuria [42]. The cost of one reagent strip and one tablet of praziquantel was US$0.06 and US$0.125 in 2013 in Malawi. After MDA in reducing the egg-positive prevalence, the positive predictive value was decreased and the negative predictive value was more than 96%. This suggests that it is practical to exclude urine occult blood-negative subjects from receiving praziquantel, and then this screening for MDA could lead to cost-effectiveness for schistosomiasis control. Since the urine reagent strips can also produce false negatives, a continuation of MDA is important for achieving better infection control.

Assuming 1810 subjects for MDA of praziquantel and presupposing the average body weight of the subject to be 40 kg, three tablets of praziquantel are needed for each subject. Under this assumption, if praziquantel is administered to all 1810 subjects, the total cost will be US$678.80, US$0.38 per person. However, assuming an egg-positive prevalence of 40% and screening using urine reagent strips and administering praziquantel, the total cost can be estimated to be US$515.90, US$0.29 per person. If the egg-positive prevalence is 40%, screening subjects for MDA using urine reagent strips, the cost reduction can be estimated to be about 24%, showing an overall cost reduction. It should be noted that in very low egg-positive prevalence settings, microhematuria is an unstable manifestation for hematobium schistosomiasis and the treatment decision should not be based on the urine reagent strips results alone [43]; different kinds of examinations for differential diagnoses should be performed according to each disease condition. Despite the reported high rate of infection noted in the previous study, the tendency to seek medication from a medical facility is not substantial, with only 34.7% of the respondents seeking treatment for hematuria at the nearest medical facility [44]. If someone has symptoms but leaves the facility, the chain of chronic/repetitive schistosome infection cannot be broken. Therefore, when symptoms such as hematuria are presented, it is important to thoroughly inform every resident that he/she should consult a medical institution promptly without neglecting it. Sustainable health education for children and young adults is the pillar for controlling schistosome infection, and it could lead to better health management.

## Conclusions

Schistosomiasis, without proper intervention and treatment, belongs to a group of diseases that could lead to morbidity and mortality to the residents in schistosome-endemic countries. And yet, schistosomiasis does not draw as much attention as malaria and tuberculosis, it is still one of the neglected tropical diseases (NTDs). Not enough countermeasures are taken to control the disease, and it could choke off economic development and growth in low-income tropical countries such as Malawi. Although it is expected that urgent schistosomiasis countermeasures will make a great social contribution in affected tropical countries, it can be easily imagined that the budgeting for countermeasures will be quite difficult in those countries. Thus, it is considered essential to prioritize to the implementation of countermeasures. School-aged children were the main targets for schistosomiasis countermeasure in the past, but this research shows that both the infection rate and the recurrence rate were higher in the labor force and that may have a direct influence on economic development. Agricultural work is the main form of labor in many tropical and subtropical areas and countries, including our target areas (Nkhotakota and Lilongwe). And contact with freshwater areas is inevitable as long as the residents are engaged in agricultural activities in these areas. Therefore, schistosomiasis should be considered to have concerning with occupational risks. Good health is positively related to economic growth or output [16]. MDA of praziquantel can assuredly reduce schistosome egg-positive prevalence in this study. The combination of MDA and urine reagent strips could be both a practical and cost-effective countermeasure for hematobium schistosomiasis. Based on the results of this study, we believe that reasonable countermeasures and well-targeted treatment could reduce the prevalence of hematobium schistosomiasis; and this could lead to an improvement in morbidity and mortality, reducing the prevalence of schistosomiasis in Nkhotakota and Lilongwe. Although the power of this study is limited, further cohort studies can be expected to provide stronger inferences about the association between schistosome infection and occupational risks. Furthermore, since schistosomiasis is presumed to have occupation-related risks, we consider that schistosome control will be a valuable step-up to economic development and make a social contribution in Nkhotakota and Lilongwe.

## Abbreviations

DOTS: Directory observed treatment, short course
MDA: Mass drug administration
NTDs: Neglected tropical diseases
